# Subtrochanteric Femur Fracture after Slipped Capital Femoral Epiphysis Pinning: A Novel Treatment

**DOI:** 10.4061/2011/809136

**Published:** 2011-01-12

**Authors:** Michael Paloski, Benjamin C. Taylor, Mark Willits

**Affiliations:** ^1^Department of Orthopedic Surgery, Doctors Hospital, 5100 West Broad Street, Columbus, OH 43228, USA; ^2^Department of Orthopedic Surgery, Grant Medical Center, 285 East State Street, Suite 500, Columbus, OH 43215, USA; ^3^Department of Orthopedic Surgery, Nationwide Children's Hospital, 479 Parsons Avenue, Columbus, OH 43215, USA

## Abstract

Slipped capital femoral epiphysis is a common injury suffered by adolescents worldwide. Treatment of most slips can be accomplished by percutaneous screw fixation, as this is an accepted and proven method associated with minimal morbidity. Complications, although limited, can be problematic for both the patient and treating physician. These include avascular necrosis, chondrolysis, infection, and fracture. We report a case of an individual who sustained a subtrochanteric femure fracture three weeks after *in situ* pinning of his left hip treated with a reconstruction intramedullary nail. This option allowed both the subtrochanteric fracture and SCFE to be treated concomitantly with minimized morbidity.

## 1. Introduction

Slipped capital femoral epiphysis (SCFE) is a common phenomenon of the proximal femur in adolescents with an unclear etiology. It is more common in boys than girls and there does seem to be some predilection to race, weight, and age [1]. The current standard for most cases of stable SCFE is *in situ* pinning and single screw fixation has shown very promising outcomes [2, 3]. However with all orthopedic implants there is a risk of peri-implant fracture and implant failure. The case presented below illustrates a patient with a slipped capital femoral epiphysis who underwent *in situ* pinning and a subsequent peri-implant fracture treated in a novel way.

## 2. Case Report

The patient was a 12-year-old, morbidly obese African-American male who presented to our outpatient clinic with a chief complaint of left hip pain for three weeks without difficulty ambulating. Anteroposterior and frog lateral radiographs were obtained at the clinic and a diagnosis of a stable, left preslip SCFE was made based on physical exam and radiographic changes of the physis. The patient was admitted to the hospital and underwent *in situ* screw fixation of his left hip the next morning with a single AO partially threaded, cannulated stainless steel screw. Intraoperatively, there were no complications and the lateral cortex was penetrated only once by the guide wire for placement of the screw. Redirection of the guide wire was necessary under fluoroscopic guidance, but this was accomplished without removing the guide pin from the single cortical hole. The patient was discharged to home care shortly thereafter with protected partial weight-bearing instructions. He followed up for his regularly scheduled postoperative appointment two weeks later and then was cleared to discontinue crutches and progress to full activity. 

**Figure 1:**
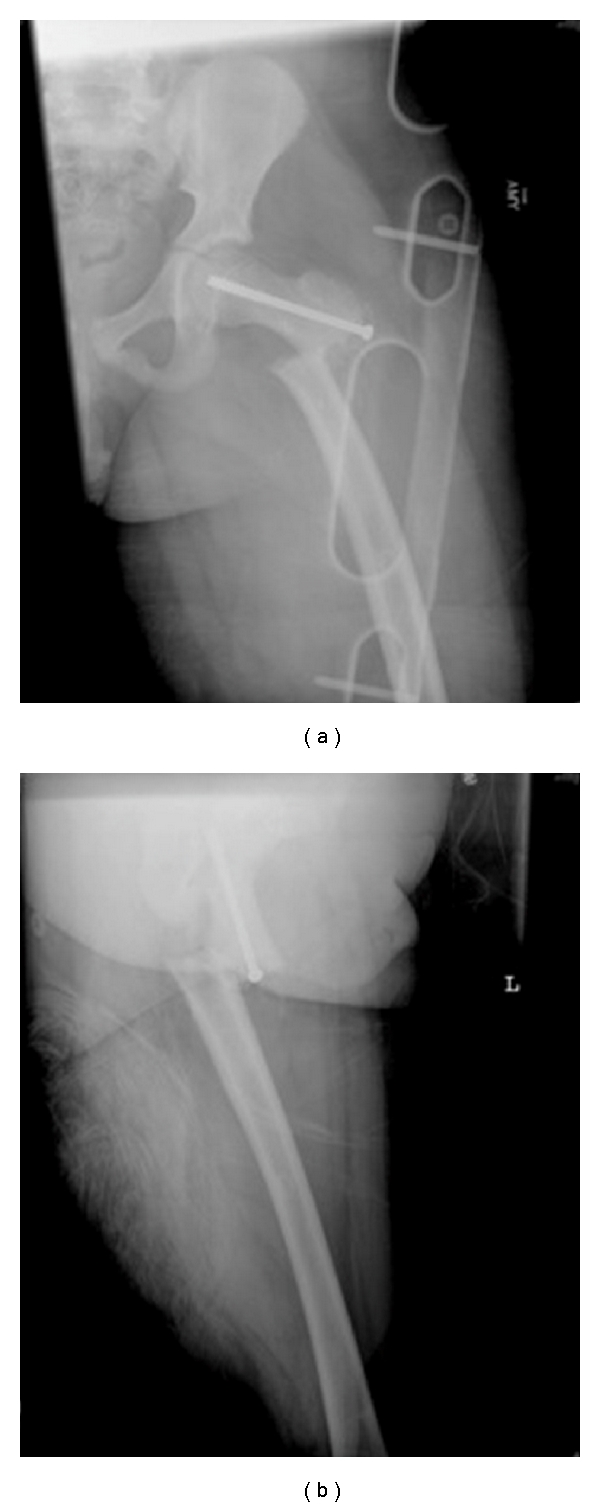
AP (a) and lateral (b) radiographic views of the left hip and thigh after the patient fell off his bicycle.

**Figure 2:**
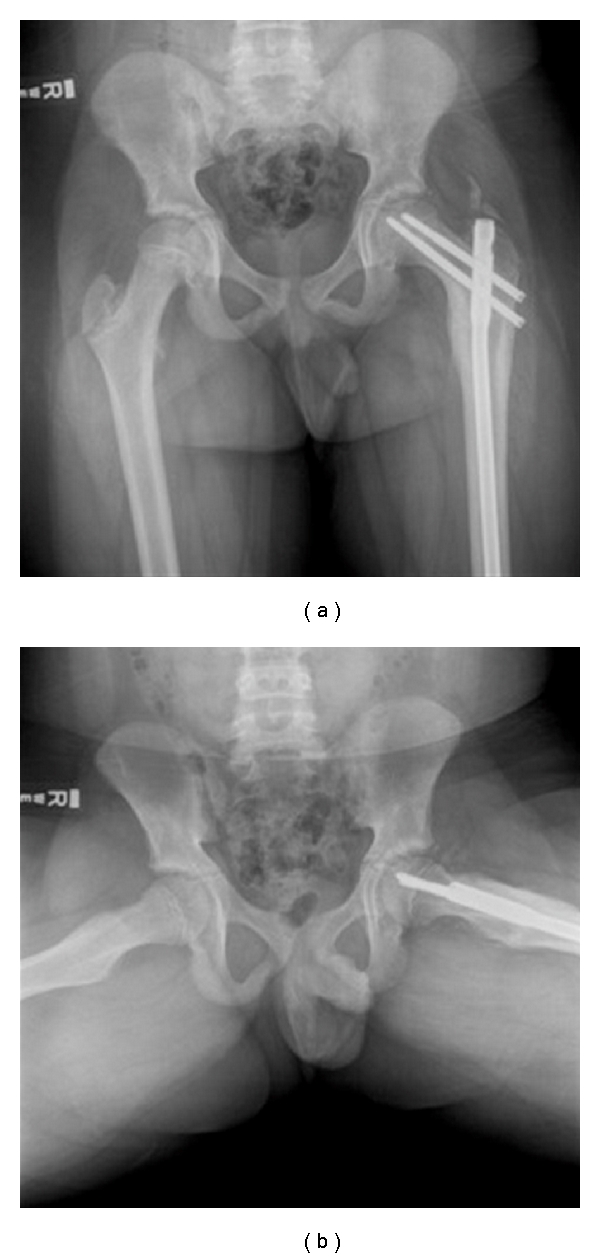
Radiographs 7 months after intramedullary fixation show a healed subtrochanteric fracture and no evidence of progression of the slip.

Approximately one month after-surgery, the patient returned to the emergency department complaining of severe left hip pain after crashing his bicycle at a high speed into a parked car. The patient was evaluated and diagnosed with a closed left subtrochanteric femur fracture (Figure 1). He was admitted to the hospital and was taken to the operating room for stabilization the following morning. The patient was placed supine on fracture table, his cannulated screw was initially removed without difficulty or complication, and subsequently a cephalomedullary nail was placed to stabilize the subtrochanteric fracture. His recovery was uneventful, and postoperative radiographs taken seven months after surgery are shown in Figure 2. No evidence of leg length discrepancy, abnormal limb rotation, or hip pathology was evident at last followup.

## 3. Discussion

*In situ *pinning of stable SCFE with single screw fixation is well documented in the literature to produce good clinical outcomes [2, 3]. The surgical technique is thoroughly reported in the literature [2, 4, 5]. Although *in situ* pinning is a commonly performed procedure with good results, it should not be approached nonchalantly as complications after fixation of SCFE are not uncommon. Reports of avascular necrosis, chondrolysis, and fracture are noted in the literature [6]. In Riley's review of his SCFE patients, eighteen percent of the patients in their study had to undergo an additional operation directly related to the complication(s) [6]. Complications related to hardware removal in SCFE are also well documented and can reach up to 34% incidence [7]. 

An infrequent complication is fracture around the implant, with subtrochanteric fractures reported to occur at a rate of only 0.3% [6]. Even in adults, subtrochanteric fracture after cannulated screw fixation of femoral neck fractures is a rare complication, with reported rates around 3% [8]. Our case is an example of a fracture that appeared to occur at the level of entry of the screw while the implant was still in place. The reason we believe reports of this in the pediatric literature are rare is because of the variability of starting points and placement of the screws for the slip. The proper starting point varies with the degree of the slip to ensure center-center penetration of the physis in both the anteroposterior and lateral planes. Therefore, entry of the screw on the anterior femoral cortex would predispose a patient to a femoral neck fracture, whereas entry on the lateral cortex would predispose a patient to a subtrochanteric fracture. 

A study by Canale et al. documented two cases of displaced femoral neck fractures at what they call the “bone-screw interface” [9]. They documented one case in which the fracture was treated by cannulated screws and Knowles pins and another case treated with a sliding hip screw. Their original screws entered the anterior femoral cortex, however, and they hypothesized that this entry point made the neck more susceptible to stress risers. Our patient presented with a preslip, and the appropriate starting point to attain perpendicular physeal penetration was on the lateral femoral cortex at the level of the lesser trochanter. We believe this screw acted as a stress riser to normal bone that underwent abnormal loads given the patient's habitus and later mechanism of injury. 

Although a single lateral femoral hole was made with the guidewire, it is unknown as how many intraoperative readjustments were made to achieve final guidewire positioning. Multiple passes have been shown to weaken the lateral cortex and decrease the energy absorbing capacity by 55.2% and increase local stresses by a factor of 1.6 [10]. Even if a screw is placed in a relatively safe zone above the lesser trochanter, pie crusting of the cortex can weaken it enough to predispose it to failure under relatively normal loads [8]. Another study by Canale et al. reported on four cases of subtrochanteric fracture after SCFE that occurred at unused drill holes in the lateral cortex [11]. They did not recommend filling the unused holes with graft or screws, but did advocate patient education and restricted weight-bearing postoperatively. 

Another point to address in this case is the use of a reconstruction nail to address both the subtrochanteric fracture and the SCFE. Kloen et al. reported on four adult cases of subtrochanteric fracture after cannulated screw placement, treated with a 95° blade plate, dynamic hip screw, and two cemented hemiarthroplasties [8]. Karagkevrekis and Rahman reported one case of a subtrochanteric fracture three weeks after screw removal for SCFE treated with a sliding hip screw [12]. Canale et al. reported four cases of subtrochanteric fractures through unused drill holes, all subsequently successfully treated with compression hip screws [11]. Although there are likely unreported cases of this pathology, we are unable to find any literature mentioning use of the reconstruction nail to address the SCFE and subsequent subtrochanteric femur fracture.

Subtrochanteric fractures of the femur are challenging due to the deforming muscular forces acting on a short, relatively mobile proximal fracture fragment. Intramedullary fixation, specifically reconstruction nails, have shown to provide optimal stabilization with up to a 95% union rate [13]. Reconstruction nails have the added benefit of fixation into the femoral head, and we were able to use this design feature to address the slip by placing the threads of the screw across the physis while concurrently stabilizing the subtrochanteric fracture with the intramedullary device. Most pediatric cases of subtrochanteric fracture are limited to sliding hip screws or blade plate devices because of the open physes. We could have chosen to use one of these devices either alone or in conjunction with an additional cannulated screw. However, in our case the patient was close enough to skeletal maturity to take advantage of the design and use of the reconstruction nail for both purposes. 

Although osteonecrosis of the femoral head is a complication with intramedullary nailing, a greater trochanteric starting point greatly diminishes this risk. It has been shown that greater trochanter apophyseodesis in patients over 10 years of age does not affect their hip function biomechanics, which makes this method of fixation for this injury more appealing [14]. Other potential complications of this technique include, but are not limited to malunion, nonunion, and intraoperative fracture. However, with proper patient selection and surgical technique, intramedullary nailing in these cases may be considered.
